# MUC1 and MUC16: critical for immune modulation in cancer therapeutics

**DOI:** 10.3389/fimmu.2024.1356913

**Published:** 2024-02-01

**Authors:** Xinyi Chen, Ineza Karambizi Sandrine, Mu Yang, Jingyao Tu, Xianglin Yuan

**Affiliations:** Department of Oncology, Tongji Hospital, Tongji Medical College, Huazhong University of Science and Technology, Wuhan, Hubei, China

**Keywords:** mucins, MUC1, MUC16, immunotherapy, tumor microenvironment, targeted drug therapy

## Abstract

The Mucin (MUC) family, a range of highly glycosylated macromolecules, is ubiquitously expressed in mammalian epithelial cells. Such molecules are pivotal in establishing protective mucosal barriers, serving as defenses against pathogenic assaults. Intriguingly, the aberrant expression of specific MUC proteins, notably Mucin 1 (MUC1) and Mucin 16 (MUC16), within tumor cells, is intimately associated with oncogenesis, proliferation, and metastasis. This association involves various mechanisms, including cellular proliferation, viability, apoptosis resistance, chemotherapeutic resilience, metabolic shifts, and immune surveillance evasion. Due to their distinctive biological roles and structural features in oncology, MUC proteins have attracted considerable attention as prospective targets and biomarkers in cancer therapy. The current review offers an exhaustive exploration of the roles of MUC1 and MUC16 in the context of cancer biomarkers, elucidating their critical contributions to the mechanisms of cellular signal transduction, regulation of immune responses, and the modulation of the tumor microenvironment. Additionally, the article evaluates the latest advances in therapeutic strategies targeting these mucins, focusing on innovations in immunotherapies and targeted drugs, aiming to enhance customization and accuracy in cancer treatments.

## Introduction

Mucins, categorized as high molecular weight glycoproteins, are divided into two subfamilies — secretory and transmembrane — based on molecular structures (1). Extensive glycosylation and growth factor-like domains in the C-terminal region, key attributes of mucins, are crucial for modulating cell surface microenvironments and enhancing interactions with other cellular receptors (2). Widely expressed on various epithelial cell surfaces, mucins undertake numerous physiological roles, including immune protection and regulation of signal transduction and transcription (3). A substantial body of research has demonstrated a strong association between the overexpression and abnormal glycosylation of mucins in various epithelial cancers with the processes of cancer cell proliferation, invasion, and metastasis (4–6). Dysregulated expression and glycosylation of MUC1 and MUC16 in cancer, notably affecting immune modulation and metastasis, impair dendritic cell function, resulting in heightened immunosuppression and advancing tumor progression (7). The comprehensive delineation of mucin dysregulation in cancer now leads to a focused analysis of MUC1 and MUC16, examining their intricate molecular interactions and pivotal influence in oncological processes.

Mucin 1 (MUC1) is a heterodimeric transmembrane glycoprotein, characterized by its polar distribution and function in providing a protective barrier (8). Its structure includes a highly glycosylated N-terminal subunit and the potentially oncogenic MUC1 C-terminal (MUC1-C), comprising a 58-amino acid extracellular domain, a 28-amino acid transmembrane region, and a 72-amino acid disordered cytoplasmic tail (9). Under normal physiological conditions, MUC1 serves as a protective barrier against external insults such as microbes, toxins, and mechanical stress, while also repairing damaged epithelium through mechanisms like epigenetic reprogramming and epithelial-mesenchymal transition (EMT), thus maintaining the stability of the epithelial layer (10). During tumorigenesis and progression, significant changes occur in the expression and function of MUC1, with over 90% of breast cancer cases exhibiting its abnormal overexpression (11, 12). In tumor cells, heightened MUC1 expression, combined with reduced glycosylation and altered polar distribution, modifies interactions with cell surface receptors (13). MUC1-C participates in several key biological processes in cancer cells, involving inflammation, proliferation, EMT, epigenetic reprogramming, and chromatin remodeling, thereby promoting cellular plasticity (14). Additionally, MUC1 enhances the stemness of cancer cells, primarily by activating pluripotency networks and also playing a role on the outer mitochondrial membrane, reducing drug-induced mitochondrial pro-apoptotic factor release and apoptosis (15, 16). Recent studies in pancreatic cancer treatment demonstrate that targeting tumor-associated MUC1 (tMUC1) with the monoclonal antibody TAB004 overcomes anoikis resistance by specifically binding to tMUC1, reducing cancer cell viability and promoting its degradation (17). Research has also indicated that MUC1 can induce chemoresistance, especially by promoting the accumulation of cancer stem cells in cervical cancer (18, 19). As an oncogenic driver, MUC1 increases the aggressiveness, metastatic potential, and drug resistance of cancer by promoting tumor cell proliferation (20, 21), EMT (22), and epigenetic changes (23, 24), and facilitates immune evasion through interactions with immune cells in the tumor microenvironment (25).

Mucin 16 (MUC16), known as the largest transmembrane mucin, has its secreted form, Cancer Antigen 125 (CA125), recognized as a serum biomarker for the diagnosis and poor prognosis of gynecological malignancies (26, 27). MUC16 is highly expressed in various epithelial cancers (28) and is considered to have a barrier function in certain organs (29, 30). Proteolytic cleavage of MUC16 releases CA-125 into the bloodstream, retaining the membrane-associated C-terminal on the cell surface; CA-125, a biomarker for ovarian cancer (31), is targeted by emerging therapies such as Chimeric Antigen Receptor (CAR) T cells (32) and antibody-drug conjugates (ADCs) (33). Approximately 80% of ovarian cancers exhibit MUC16 expression, which is present in all ovarian cancer subtypes (including serous, mucinous, endometrioid, and clear cell) though it varies in expression (34). Recent studies suggest that MUC16 may play a role in modulating immune responses in different cancers. Gubbels JA et al. reported that MUC16 can protect ovarian cancer cells from NK cell attacks by inhibiting the formation of immune synapses between NK cells and ovarian cancer cells (35). Belisle JA et al. found that MUC16 can bind to NK cells, B cells, and monocytes through Siglec-9, which, as an inhibitory receptor, can weaken the function of T cells and NK cells (36). Other research reveals that the proportion of circulating regulatory T (Treg) cells is related to the level of CA125 in the serum, and the C-terminal of MUC16 can activate the JAK2/STAT3 signaling pathway triggered by tumor-secreted IL-6, promoting the expression of Foxp3 and the accumulation of tumor-associated Tregs in pancreatic cancer (37). Additionally, studies show that inflammatory cytokines TNFα and IFNγ can stimulate MUC16 expression in breast cancer, endometrial cancer, and ovarian cancer cells via NFκB, and in these cancer tissues, increased MUC16 expression is associated with elevated cytokine levels (38). Inflammatory stimuli such as oxidative stress and treatment with cytokines IFNγ, IL-1α, TNFα have also been found to alter the glycosylation pattern of MUC16 in pancreatic cancer cells (39), suggesting that MUC16 may play a key role in promoting inflammatory signaling in cancer.

In summary, MUC1 and MUC16, key molecules within the mucin family, exhibit roles in cancer biology surpassing the conventional barrier formation in normal epithelial cells. These mucins contribute to various aspects of tumor development, affecting tumor cell proliferation, survival, migration, and interactions with the immune system. The roles of these mucins in tumor immune surveillance and as potential therapeutic targets present new opportunities for cancer treatment. This review examines the roles of MUC1 and MUC16 in cancer immune regulation and therapeutic effectiveness, exploring their potential in precision medicine. Challenges currently faced and future research directions are also discussed, offering novel insights and strategies for cancer therapy.

## The relevance of MUC1 and MUC16 to tumor development and progression

Extensive research has established MUC1 and MUC16 as key players in cancer development, with expression patterns significantly influencing tumor progression and serving as vital prognostic markers. Specifically, research by Ren et al. noted an increase in MUC1 expression across various tumors, enhanced by heregulin, which positions its C-terminal subunit in mitochondria, thereby inhibiting cisplatin-induced apoptotic signaling and reducing tumor cell sensitivity to chemotherapy drugs (21). Rajabi et al. showed that MUC1-C protein, by augmenting the activity of DNMT1 and DNMT3b, induces high levels of DNA methylation, leading to the silencing of tumor suppressor genes and thus exerting its carcinogenic effect (23). Moreover, MUC1-C’s role in cancer progression is further confirmed by enhancing the binding of BMI1 to the CDKN2A promoter and suppressing the expression of tumor suppressor genes like p16INK4a (24). In ovarian cancer research, Ma et al. identified MUC1 as a key regulatory factor in the disease’s development, primarily through upregulating EGFR expression and activating the AKT signaling pathway to promote tumor cell proliferation, while also highlighting Taxol’s significant therapeutic potential in targeting ovarian cancers with abnormally high MUC1 expression (40). Zong et al. indicated that circWHSC1, by binding with miR-145 and miR-1182, upregulates MUC1 expression in ovarian cancer, promoting cell proliferation and metastasis (41). MUC16 plays a significant role in ovarian cancer research, with its relationship to tumor growth, metastasis, and chemotherapy resistance extensively supported by research. Liu et al. found that the inflammatory microenvironment in ovarian cancer activates the MUC16 gene via the NF-κB pathway, leading to elevated CA125 levels, thereby exacerbating tumor progression and invasiveness (42). Furthermore, Wang et al. found that MUC16’s regulatory effect on GLUT1 expression might be a contributing factor to the proliferation and progression of ovarian epithelial cancer (43). In terms of tumor metastasis, Huo et al. noted that the CA125/MUC16 complex significantly enhances tumor cell migration and metastasis capabilities by downregulating DKK1 expression and activating the SGK3/FOXO3 pathway (44). Concurrently, Gubbels et al. demonstrated that MUC16’s high-affinity binding with mesothelin, mediated by N-glycan-dependent interactions, is crucial for peritoneal metastasis of ovarian tumors (45). Research by Matte et al. indicated that MUC16 can weaken TRAIL-induced apoptosis, a function related to its role in tumor formation and chemotherapy resistance (46).

The impact of MUC1 and MUC16 on the progression of breast cancer has been extensively studied and confirmed. Woo et al. showed that in breast cancer, abnormal overexpression of MUC1 promotes VEGF production by activating the AKT signaling pathway, a mechanism playing a vital role in tumor angiogenesis, growth, and metastasis (47). MUC1 also enhances the invasiveness of breast cancer cells by disrupting ATAD3A and activating Pink1-related mitochondrial autophagy, revealing the critical role of the MUC1/ATAD3A/Pink1 axis in breast cancer progression (48). Additionally, in HER2-positive breast cancer, Raina et al. revealed the potential efficacy of MUC1-C targeted therapy in reducing HER2 activity and reversing trastuzumab resistance, highlighting the therapeutic value of MUC1-C inhibitors (49). MUC1 glycosylation modifications also regulate the chemosensitivity of breast cancer cells, with Xi et al. confirming that knocking out MUC1 or removing specific glycosylation modifications enhances chemotherapy effects (50). In triple-negative breast cancer (TNBC) research, Yamashita et al. confirmed that MUC1-C promotes cancer progression by activating cytoplasmic nucleotide pattern recognition receptors (PRRs) such as RIG-I, MDA5, and cGAS, and the cGAS-STING pathway (51). Regarding MUC16, research by Chaudhary et al. found that its high expression in TNBC is associated with enhanced action of the RNA-binding protein ELAVL1/HuR, promoting TNBC metastasis to the lungs through regulation by the HuR/cMyc axis (52). Research by Lakshmanan et al. revealed that MUC16 overexpression in breast cancer alters interactions with JAK2, accelerating the G2/M phase transition of the cell cycle to promote cellular proliferation and apoptosis resistance, while its silencing induces cell cycle arrest and enhances TRAIL-mediated apoptosis (53). Additionally, the CDK4/6 inhibitor palbociclib, by downregulating MUC16 expression, exerts an inhibitory effect on the expression of key genes in the breast cancer cell cycle and suppresses ER(-) aggressive breast cancer cells (54). In cervical cancer research, Lv et al. found that the EGFR-TKI drug erlotinib, targeting MUC1-positive cervical cancer through the MUC1-EGFR-CREB/GRβ-IL-6 axis, significantly inhibits taxol-resistant tumor stem cells and reduces residual tumor stem cells after chemotherapy, offering the potential to improve patient prognosis (19).

The impact of MUC1 and MUC16 in lung cancer development has been further substantiated. Research by Raina et al. showed that MUC1-C activates the PI3K/Akt/mTOR pathway in non-small cell lung cancer (NSCLC) by interacting with PI3K p85, promoting tumor cell proliferation and significantly correlating with poor patient prognosis (55). Overexpression of MUC16 in lung cancer enhances tumor growth and migration, while also influencing development and metastasis through the JAK2/STAT3/GR axis, potentially contributing to chemotherapy resistance to agents like cisplatin and gemcitabine (56). Lei et al. disclosed that in lung cancer, ERO1L promotes IL6R secretion to activate the NF-κB pathway, leading to an upregulation of MUC16 gene expression, and the C-terminal of MUC16 further fosters the EMT phenotype and IL6 release, creating a positive feedback loop that exacerbates lung cancer progression (57). Research by Kanwal et al. also indicated that air pollution is associated with elevated MUC16 mRNA levels in lung cancer samples, and mutations in the MUC16 gene might enhance its expression, thereby increasing tumor resistance to chemotherapy and invasiveness (58).

MUC1 and MUC16 also play pivotal roles in gastrointestinal cancers. In colorectal cancer research, Morimoto et al. discovered that BRAF(V600E) mutant colorectal cancer exhibits overexpression of MUC1 protein, which, by activating the SHP2 phosphatase, further strengthens RAS/ERK signaling, leading to increased tumor cell proliferation and resistance to BRAF inhibitors; targeting MUC1-C could potentially enhance treatment sensitivity and inhibit tumor growth (59). In the progression from colitis to colorectal cancer, targeted inhibition of MUC1-C plays a crucial role in blocking LGR5+ intestinal stem cells (ISCs), Myc, and pluripotency factors signaling pathways, slowing down the worsening of colitis (10). Conversely, in liver cancer cell lines HepG2 and Huh7, Huang et al. found that MUC16 gene knockdown significantly enhances cell migration and invasion capabilities, with RNA-seq analysis confirming the molecular basis of these changes (60). Additionally, the role of MUC1 and MUC16 in pancreatic cancer progression has been extensively validated. Shukla et al. demonstrated that in pancreatic cancer cells, MUC1’s interaction with HIF-1α promotes glycolysis, leading to increased dCTP levels and decreased gemcitabine efficacy, while targeting HIF-1α markedly reduces tumor burden, underscoring MUC1’s essential role (61). Furthermore, Behrens et al. found that the cytoplasmic tail of MUC1 (MUC1.CT) regulates genes associated with invasion, angiogenesis, and metastasis, enhancing the tumor microenvironment’s carcinogenic properties (62). On the other hand, threonine deficiency and tRNA synthetase inhibition can reduce MUC1 levels in pancreatic cancer cells, thereby inhibiting tumor cell migration (63). Roy et al. further observed that in pancreatic cancer, MUC1 overexpression induces EMT, enhancing tumor cell invasion and metastasis through MUC1’s interaction with β-catenin, leading to its nuclear translocation and activation of genes associated with EMT and metastasis (22). Additionally, it has been discovered that in pancreatic ductal adenocarcinoma (PDAC), MUC1 overexpression induces non-canonical TGF-β signaling, where MUC1 overexpression activates the JNK pathway in response to TGF-β, thus altering its function from a tumor suppressor to a tumor promoter (64). In PDAC, MUC16’s role is also significant. Thomas et al. reported that in PDAC patients, high MUC16 protein expression correlates with faster tumor progression, more metastasis, aggressive subtypes like basal-like and squamous tumors, and shorter survival, with its abnormally glycosylated form activating AKT and GSK3β signaling pathways, thereby exacerbating tumor malignancy (65). Chirravuri-Venkata et al. showed that MUC16 overexpression in PDAC is closely associated with high tumor antigen load, polarization of heterogenous stromal fibroblasts (CAFs), and a significant increase in TP53 gene deletion risk, collectively indicating a poor prognosis (66). Marimuthu et al. revealed that MUC16 activates the JAK2/STAT1 signaling pathway, promoting liver metastasis in PDAC (67). Rajesh et al. revealed that in PDAC, truncated O-glycan forms of MUC16 interact with the α4β1 integrin complex, activating the ILK/FAK signaling pathway and enhancing tumor invasiveness (68). Muniyan et al. indicated that MUC16 significantly promotes PDAC proliferation and migration by interacting with extracellular matrix proteins like selectin-3 and endothelin and through FAK-mediated signaling pathways (69). Shimizu et al. confirmed that high expression of MUC16 in PDAC correlates with tumor invasiveness. MUC16 enhances pancreatic cancer cell invasion and migration by interacting with endothelin, thus playing a key role in the progression and worsening of PDAC (70). In gallbladder cancer research, Fan et al. established that the MUC16 carboxy-terminal peptide, by interacting with stathmin1, enhances the migration and invasion capabilities of gallbladder cancer cells (71). Deng et al.’s study revealed a positive correlation between GATA6 and EMT markers in cholangiocarcinoma, regulating β-catenin’s nuclear translocation through its downstream target MUC1, thereby promoting EMT and metastasis, indicating the critical role of the GATA6/MUC1/β-catenin signaling pathway in cholangiocarcinoma progression (72).

In prostate cancer, MUC1-C has been shown to activate the BAF complex in prostate cancer stem cells, enhancing the expression of NOTCH1 and NANOG, thus promoting self-renewal of tumor stem cells and accelerating the progression of neuroendocrine prostate cancer (15). Research by Shigeta et al. indicated that in urothelial cancer, increased activity of MUC1-C enhances resistance to cisplatin, while inhibition of MUC1 can restore drug sensitivity, a process involving regulation of the PI3K-AKT-mTOR pathway and ROS (73).

**Figure 1:**
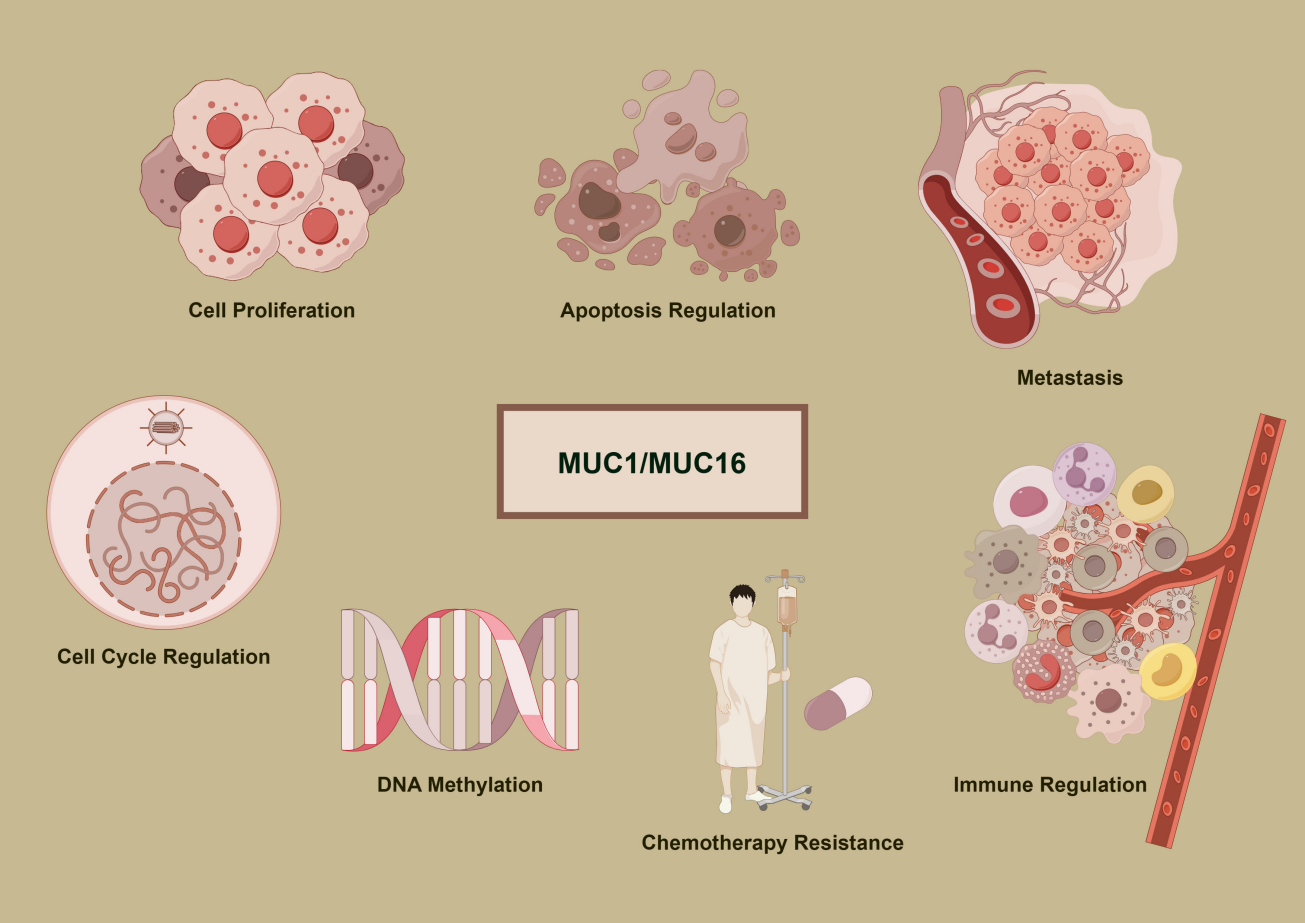
Oncogenic mechanisms of MUC1 and MUC16. The diagram delineates the complex functions of MUC1 and MUC16 in cancer mechanisms, illustrating their roles in promoting cell division, inhibiting apoptosis, facilitating metastasis, modifying DNA methylation patterns, conferring chemoresistance, and regulating immune interactions, which are pivotal in the progression of cancer and the response to therapies.

**Figure 1** delineates the multifunctional roles of MUC1 and MUC16 in cancer development. Accompanying **Tables 1** and **2** detail these mucins’ expression and functions across cancer types, underlining their importance in disease mechanisms and as potential therapeutic targets.

**Table 1:** Expression and oncogenic roles of MUC1 in various human cancers.

Cancer Classification	Expression Status	Biological roles	Target Genes & Interacting Proteins	References
Various Carcinomas	Upregulated	Blocks Intrinsic Apoptotic Pathway; Mitochondrial Localization	c-Src, HSP70, HSP90, ErbB Receptors	(16)
Various Carcinomas	Upregulated	Induces Acquired Chemoresistance	ABCB1, EGFR	(18)
Breast Carcinomas and Others	Upregulated	Induces Expression of DNMT1 and DNMT3b, Affects DNA Methylation	DNMT1, DNMT3b, NF-κB p65	(23)
Colon, Lung, and Breast Carcinomas	Upregulated	Attenuates Apoptotic Response; Confers Resistance to Genotoxic Agents	MUC1-C Subunit	(21)
Ovarian Cancer	Upregulated	Mediates Tumor Growth, Increases EGFR Expression, Influences AKT Pathway	EGFR, AKT Pathway Components	(40)
Ovarian Cancer	Upregulated	Increases Cell Proliferation, Migration, Invasion; Inhibits Apoptosis	Regulated by circWHSC1 via miR-145 and miR-1182 Sponging	(41)
Breast Cancer	Upregulated	Promotes Mitophagy, Oncogenicity via Mitochondrial Interaction and ATAD3A Degradation	Interacts with ATAD3A, Protects Pink1	(48)
Breast Cancer	Upregulated	MUC1 Contributes to Drug Insensitivity; O-Glycosylation of MUC1-N Decreases Drug Permeation	GCNT3 (Related to MUC1 O-Glycosylation)	(50)
Breast Cancer	Upregulated	Promotes VEGF Synthesis and Secretion; Accelerates Tumor Growth and Angiogenesis	Influences AKT Signaling and Insulin-Like Growth Factor-1 Receptor Activation	(47)
HER2-Positive Breast Cancer	Upregulated	Associates with HER2; Promotes HER2 Activation; Contributes to Trastuzumab Resistance	Interacts with HER2; Affects p-HER2 and AKT Activation; Implicated in Phospho-p27 and Cyclin E Regulation	(49)
Cervical Cancer	Upregulated	Induces CSC Enrichment via EGFR Activation	EGFR, IL-6, CREB, GRβ	(19)
Colorectal Cancer	Upregulated	Drives Stemness; Influences ISC Population, Myc Induction, Progression of Colitis to CRC	Interacts with MYC, LGR5, BMI1, ALDH1, FOXA1, LIN28B, OCT4, SOX2, NANOG	(10)
Colorectal Cancer	Upregulated	Promotes CRC Progression; Drives Cell Cycle Progression via MYC; Enhances RAS→ERK Signaling through SHP2 Activation	Activates MYC and NOTCH1; Associates with SHP2	(59)
Pancreatic Ductal Adenocarcinomas	Upregulated	Induces EMT, Increases Invasiveness	β-Catenin, Vimentin, Slug, Snail, E-Cadherin	(22)
Pancreatic Cancer	Upregulated	Contributes to Gemcitabine Resistance via Stabilizing HIF-1α	HIF-1α; Impacts Pyrimidine Biosynthesis Genes (TKT, CTPS)	(61)
Cholangiocarcinoma	Upregulated	Facilitates EMT and Metastasis via β-Catenin Pathway	GATA6, β-Catenin	(72)
Urothelial Carcinoma	Upregulated	Contributes to Cisplatin Resistance; Regulates MDR1, PI3K-AKT-mTOR Pathway, and xCT Stabilization	MDR1, PI3K-AKT-mTOR Pathway, xCT	(73)
Neuroendocrine Prostate Cancer	Upregulated	Activates BAF Complex, Drives Stemness	E2F1, NOTCH1, NANOG	(15)

**Table 2:** Expression and oncogenic roles of MUC16 in various human cancers.

Cancer Classification	Expression Status	Biological roles	Target Genes & Interacting Proteins	References
Breast, Endometrial, Ovarian Cancers	Upregulated	Responds to TNFα and IFNγ; Enhanced Expression and Distribution Alteration in Cancer	NFκB (Regulates Cytokine-Induced Expression)	(38)
Ovarian Cancer	Upregulated	Expression Enhanced in Response to Inflammation, Contributing to Tumor Progression	NF-κB (Activates MUC16 Gene Expression)	(42)
Epithelial Ovarian Cancer	Upregulated	Promotes Cell Proliferation and Disease Progression by Regulating Glucose Metabolism	Regulates GLUT1 Expression, Controlling Glucose Uptake	(43)
Epithelial Ovarian Cancers	Upregulated	Attenuates TRAIL-Induced Apoptosis; Inhibits Activation of Caspase-8 and Mitochondria	Decreases TRAIL Receptor R2 (DR5) Expression; Influences cFLIP mRNA Levels and Degradation	(46)
Ovarian Cancer	Upregulated	Facilitates Tumor Metastasis via SGK3/FOXO3 Pathway; Reduces DKK1 Expression	Binds Mesothelin, Regulates DKK1 and SGK3/FOXO3 Pathway	(44)
Ovarian Cancer	Upregulated	Facilitates Peritoneal Metastasis Through Interaction with Mesothelin	Binds to Mesothelin; N-Glycan Dependent Interaction	(45)
Breast Cancer	Upregulated	Promotes Proliferation and Anti-Apoptosis; Induces G2/M Transition	Interacts with JAK2; Influences STAT3 and c-Jun Activation, Cyclin B1, Aurora Kinase A, TRAIL	(53)
Triple-Negative Breast Cancer	Upregulated	Promotes TNBC Lung Metastasis; Facilitates Invasion, Migration, and Colony Formation	Interacts with ELAVL1/HuR; Regulates cMyc Expression	(52)
Lung Cancer	Upregulated	Enhances Metastasis via IL6 Signaling and EMT Promotion	ERO1L, IL6R, NF-κB	(57)
Lung Cancer	Upregulated	Regulates TSPYL5, Promotes Cell Growth, Migration, Chemoresistance; Suppresses p53	TSPYL5, JAK2, STAT3, GR	(56)
Pancreatic Ductal Adenocarcinoma	Upregulated	Enhances Tumor Malignancy via AKT and GSK3β Signaling; Interacts with EGF Receptors	Epidermal Growth Factor (EGF)-Type Receptors	(65)
Pancreatic Ductal Adenocarcinoma	Upregulated	Promotes Cell Survival, Colonization, and Metastasis to Liver	Alters Neuropilin-2 (NRP2) via JAK2/STAT1 Signaling	(67)
Pancreatic Ductal Adenocarcinoma	Upregulated	Enhances Tumor Malignancy; Activates ILK/FAK-Signaling Axis; Increases Tumor Cell Migration	Integrin Complexes (α4β1); Involved in FAK Signaling	(68)
Pancreatic Ductal Adenocarcinoma	Upregulated	Promotes Cell Proliferation, Colony Formation, Migration; Facilitates Tumor Growth and Metastasis	Interacts with Galectin-3, Mesothelin, FAK; Modulates Tumor-Associated Carbohydrates, Akt, ERK/MAPK Signaling	(69)
Gallbladder Cancer	Associated with Metastasis	MUC16 C-Terminal Fragment Promotes Cell Migration and Invasion	Interacts with Stathmin1, Affecting its Phosphorylation and Tubulin Binding	(71)

## The role of MUC1 and MUC16 in the tumor immune microenvironment

In tumor immune microenvironment research, the roles of MUC1 and MUC16 in regulating mechanisms by which tumor cells evade immune surveillance, and how these proteins influence immune escape and treatment response through different pathways, are currently focal points of investigation. Madsen et al. highlighted the role of MUC1 and MUC16 in breast and pancreatic cancer cells, showing that reducing their expression through COSMC gene knockdown enhances tumor cell sensitivity to natural killer cell-mediated antibody-dependent cellular cytotoxicity (ADCC) and cytotoxic T lymphocyte (CTL) killing. This suggests that high expression of MUC1 and MUC16 aids tumor cell immune evasion (74). Additionally, research by Menon et al. showed that MUC1 and MUC16 inhibit Toll-like receptor (TLR)-mediated innate immune responses, reducing expression of inflammatory factors IL-6, IL-8, and TNF-α, potentially linked to tumor immune escape (75). For MUC1, Beatson et al. revealed that tumor cell surface tumor-specific MUC1 glycoforms (MUC1-ST) bind to Siglec-9, activating myeloid cells to secrete key factors influencing tumor progression, induce macrophages to transform into tumor-associated macrophage (TAM) phenotypes, and increase PD-L1 expression (25). MUC1’s regulatory effect on the immune environment includes inhibiting myeloid-derived suppressor cells (MDSCs) proliferation and immunosuppressive functions, where its absence in mice leads to increased MDSCs numbers and activity, promoting tumor growth, and MUC1 deletion also heightens MDSCs’ release of factors such as iNOS, ARG1, and TGF-β, further inhibiting T cell activity (76). Chan et al. found that soluble MUC1 inhibits T cell proliferation and function, likely by causing T cell arrest at the G(0)/G(1) phase of the cell cycle, thus impeding T cell activation and playing a key role in tumor immune evasion (77). MUC16’s role in the tumor immune microenvironment is equally significant. Zhang et al.’s analysis of 10,195 solid tumor patients showed that MUC16 mutations correlate with a higher tumor mutational burden (TMB) and neoantigen load, increased CD8A and PD-L1 expression in the tumor immune microenvironment, and are linked to improved patient survival and clinical response rates, highlighting their significance as genomic markers in evaluating response to immune checkpoint inhibitors (ICIs) therapy (78). TNFα and IFNγ enhance MUC16 expression in breast, endometrial, and ovarian cancer cells through the NFκB pathway, with this upregulation linked to immune regulatory factor activity, indicating that MUC16 modulation may benefit treatment (38).

In ovarian cancer immunoregulation research, MUC16 plays a critical role, as evidenced by studies. Gubbels et al. confirmed that MUC16, as a highly glycosylated molecule expressed on the surface of ovarian cancer cells, promotes tumor immune evasion by blocking the establishment of immune synapses between NK cells and tumor cells, providing a selective survival mechanism for tumor metastasis (35). Similarly, Belisle et al. demonstrated that MUC16 binds with Siglec-9 on NK cells, B cells, and monocytes, inhibiting their functions and further facilitating immune escape, growth, and metastasis of ovarian cancer (36). Wu et al. also observed in ovarian cancer research that MUC16 activates neutrophils’ Siglec-9 receptors, inducing inflammatory and immunosuppressive characteristics in these cells and weakening NK cells’ killing ability, collectively promoting tumor immune evasion (79). Further, Belisle et al. found in ovarian cancer that MUC16 binding with a specific CD16(+) CD56(dim) NK cell subset induces their transition from an active to a passive or suppressed state, further aiding immune evasion in ovarian cancer (80). Felder et al. showed that in ovarian cancer, MUC16 is crucial for maintaining cytotoxicity of natural killer and macrophage cells, with its absence reducing these immune cells’ cytotoxic effects and increasing tumor cell sensitivity to ADCC, thereby prolonging survival in a mouse model (81). Long-term monitoring of ovarian cancer patients using digital cytometry enhanced with gold nanoparticles revealed that elevated MUC16 levels on peripheral blood mononuclear cells (PBMCs) surface predict tumor relapse and metastasis risk, with MUC16 levels in ovarian cancer patients’ PBMCs higher than in healthy controls (82). According to Zhai et al., overexpression of MUC16 in ovarian cancer activates the PI3K/AKT pathway, promoting tumor cell proliferation and invasion while also enhancing anti-tumor immune responses, primarily manifested in dendritic cell maturation and CD8+ T cell activation (83). Patankar et al. revealed that ovarian tumor marker CA125 (MUC16), through its specifically expressed oligosaccharides, inhibits NK cell cytotoxicity, significantly reducing their cytotoxicity and playing a key role in the immune evasion mechanism of ovarian tumors (84). Additionally, Winkler et al. discovered a significant negative correlation between iNKT+/CD3+/CD161+ lymphocytes in ovarian cancer patient tumor tissues and serum CA125 concentration, suggesting a potential role of CA125 in modulating immune cells in the tumor microenvironment (85). Innovations in ovarian cancer treatment targeting MUC16 have shown potential. Koneru et al. developed 4H11-28z CAR T cells targeting the MUC16ecto antigen, enhancing these CAR T cells’ immune response and tumor elimination capabilities against ovarian cancer by co-expressing IL-12 (86). Similarly, Li et al. employed PD1-antiMUC16 dual-target CAR-T cells to treat epithelial ovarian cancer, demonstrating *in vivo* that these dual-target CAR-T cells exhibited greater killing efficacy compared to their single-target counterparts and significantly prolonged survival in a mouse model, underscoring MUC16’s central role in immunotherapy (87). Additionally, Crawford et al. demonstrated that the dual-specific antibody REGN4018, targeting highly expressed MUC16 in ovarian cancer, effectively activates T cells and kills MUC16-positive tumor cells *in vitro*, with preclinical animal model studies revealing that its combination with anti-PD-1 antibodies significantly enhances anti-tumor effects (88). Boland et al. found that in epithelial ovarian cancer patients receiving immune checkpoint inhibitor therapy, increased serum MUC16 levels correlated with poor immunotherapy outcomes, suggesting its prognostic indicator value (89). Similarly, Baert et al. indicated that in high-grade serous ovarian cancer patients’ serum samples, an increase in MUC16 was positively correlated with the rise of immunosuppressive factors like IL-10 and negatively correlated with overall survival rates, suggesting MUC16 as an adverse prognostic marker (90). Kline et al. revealed that MUC16 inhibits ADCC by binding with antibodies and suppressing Fc-γ receptor activation, with patients having low MUC16 levels in recurrent platinum-sensitive ovarian cancer showing improved survival following farletuzumab treatment compared to a control group (91).

The role of MUC1 in breast cancer immunity research, particularly in modulating tumor immune escape mechanisms, has garnered significant attention. Maeda et al. showed that in TNBC, MUC1-C upregulation recruits MYC and NF-κB p65 to the PD-L1 promoter, enhancing PD-L1 transcription and leading to immune escape and reduced patient survival; targeted intervention against MUC1-C not only suppresses PD-L1 expression but also enhances infiltration and activity of CD8+ T cells in tumors (92). Beatson et al. further revealed that MUC1-ST binding to Siglec-9 promotes differentiation of monocytes into tumor-associated macrophages (TAMs), which recruit neutrophils, inhibit T cell function, and promote tumor cell invasion, closely associated with poor prognosis in breast cancer patients (93). Zhou et al. focused on MUC28z CAR T cells targeting tMUC1 in TNBC treatment, with these cells specifically recognizing tMUC1 in most TNBC subtypes and exhibiting potent targeted cytotoxicity by increasing Granzyme B and interferon-gamma (IFN-γ) production (94). Yamashita et al. further confirmed MUC1-C as a core regulatory factor of the TNBC transcriptome, playing a significant role in inducing the immunosuppressive IFN-γ pathway, with MUC1-C expression correlated with upregulation of immunosuppressive effector factors like IDO1 and COX2/PTGS2, and associated with CD8+ T cell exhaustion and dysfunction in the tumor immune microenvironment (TIME) (95). Another study revealed that high expression of MUC1-C in TNBC is closely associated with the absence of tumor-infiltrating lymphocytes (TILs), and its synergistic action with PBRM1 enhances STAT1 and IRF1 expression in the interferon pathway, affecting T and NK cell functions and promoting tumor DNA damage resistance and immune escape (96). Grosso et al. confirmed that expression of secretory mucin 1 (MUC1/sec) in breast tumor cells can initiate T cell-dependent immune rejection responses and promote the recruitment of immune cells by increasing chemokine CCL2 secretion, aiding in anti-tumor therapy (97). Lin et al.’s study on MUC1 mRNA nanoparticle vaccines for TNBC treatment demonstrated their ability to activate CTLs against MUC1-expressing tumor cells and, when used alongside CTLA-4 antibodies, reduce Tregs, thereby enhancing CTL cytotoxicity and effectively modulating the tumor microenvironment to strengthen the immune response (98). Similarly, Liu et al. demonstrated the effectiveness of using nanoparticles (NPs) to deliver MUC1 mRNA vaccines to lymph node dendritic cells (DCs) in TNBC treatment, a method that activates specific T cells and combines with anti-CTLA-4 monoclonal antibodies to enhance immune response (99). In other gynecological tumors, Hu et al. showed that MUC16 mutations improve patient prognosis by enhancing cytotoxic T lymphocyte infiltration and anti-tumor immunity in the endometrial cancer microenvironment (100). Wang et al. observed in cervical cancer that overexpression in MUC16 mutation samples is closely associated with enhanced immune cell activity in the tumor microenvironment and improved prognosis (101). In lung cancer research, Bouillez et al. found that targeting MUC1-C in NSCLC enhances CD8+ TILs’ cytotoxicity against tumor cells and plays a crucial role in promoting PD-L1 induction, aiding tumor cells in evading immune surveillance. Additionally, reducing PD-L1 expression and increasing IFN-γ levels through targeting MUC1-C bolsters the immune system’s ability to recognize and destroy tumor cells, effectively countering tumor progression (102). Concurrently, MUC1-C activates the NF-κB/ZEB1 pathway to promote PD-L1 (CD274) expression while suppressing immune effector genes like TLR9 and IFNG, thus enhancing PD-L1 expression and inhibiting immune responses, closely associated with decreased patient survival (103). Wang et al. demonstrated that dual-target Tan CAR-T cells, targeting both MUC1 and PSCA in NSCLC and used in combination with anti-PD-1 antibody therapy, exhibit superior anti-tumor effects compared to single-target CAR-T cells, with their efficacy significantly enhanced when combined with anti-PD-1 treatment (104). In NSCLC treatment, Jiang et al. discovered that evodiamine, by downregulating MUC1-C protein expression, modulates PD-L1 expression, thereby effectively inhibiting tumor growth and inducing apoptosis, while enhancing CD8+ T cells’ effector function, with its combination with anti-PD-1 monoclonal antibodies significantly bolstering tumor control (105). In MUC16-related research, Patel et al. revealed that in lung adenocarcinoma, a matrix metalloproteinase-resistant variant of MUC16 may lead to tumor cell evasion of the immune system, reducing tumor-specific peptide presentation through HLA-A and HLA-B molecules, thereby promoting tumor immune escape (106). In gastrointestinal tumor research, studies on MUC1 and MUC16 are crucial in unveiling tumor immune evasion mechanisms and progression. Saeland et al. demonstrated that abnormal glycosylation of MUC1 in colon cancer, particularly the exposure of Tn and TF antigens, may promote tumor immune evasion through binding with MGL expressed on DCs and macrophages; additionally, MUC1’s correlation with the adverse prognostic marker Helix pomatia agglutinin (HPA) indicates significant interactions between MUC1 and immune regulatory factors (107). Zhang et al. observed in the colon cancer tumor microenvironment that MUC1 presence leads to the accumulation of Tregs, MDSCs, and TAMs; blocking the PD1/PD-L1 pathway reduces immune suppressive cells in the tumor microenvironment, enhancing T cell cytotoxic responses and inhibiting tumor growth (108). In colitis-associated colorectal cancer (CAC), Sheng et al. found that abnormal overexpression of MUC1 significantly exacerbates tumor progression, while inhibiting MUC1 expression can reduce inflammation and tumor progression, marked by increased CD8+ T lymphocytes and reduced macrophages in tumors (109). Monti et al. revealed that in pancreatic cancer, MUC1 alters cytokine profiles of monocyte-derived DCs, turning them into regulatory cells with high IL-10 (an immunosuppressive cytokine) and low IL-12 (an immune-promoting cytokine), thereby inhibiting DCs’ ability to activate Th1 type immune responses and promoting tumor immune evasion (110). Beatty et al. emphasized that in pancreatic cancer precursor lesion intraductal papillary mucinous neoplasm (IPMN), abnormal expression of MUC1 promotes specific immune responses, including IgG production and T cell infiltration (111). Research on the C-terminal of MUC16 in pancreatic cancer has shown a positive correlation with Foxp3 expression, aligning with serum CA125 levels and the proportion of circulating Tregs, where MUC16c’s activation of the IL-6 JAK2/STAT3 pathway enhances Foxp3 expression, thereby promoting Treg accumulation in tumor tissue (37). In pancreatic ductal adenocarcinoma models with KrasG12D and Trp53R172H mutations, research by Lakshmanan et al. demonstrated that MUC16 deletion significantly impedes tumor progression and metastasis, leading to prolonged survival, and influences the tumor microenvironment, possibly by modulating the expression of genes like Actg2, Myh11, and Pdlim3 (112). The study by Chirravuri-Venkata et al. in PDAC indicated that MUC16, in conjunction with the TP53 family, regulates tumor-stromal heterogeneity. MUC16’s impact transcends tumor cells, prominently modulating the tumor environment by guiding the differentiation of CAFs (66). In cholangiocarcinoma research, Zhang et al. revealed that the interaction between MUC1 and EGFR activates the EGFR/PI3K/Akt pathway, leading to the accumulation of Foxp3+ Tregs, aiding tumor immune evasion and thus promoting cholangiocarcinoma development and metastasis (113).

In melanoma-related research, MUC1 and MUC16 have demonstrated distinct roles in tumor progression and immune responses. Regarding MUC1, a study by Wang et al. in a melanoma mouse model found that overexpression of TRAF6 significantly enhanced MUC1-specific Th1 and Tc1 responses while reducing the proportion of Tregs, thereby ameliorating the immunosuppressive state and inhibiting tumor growth (114). In melanoma treatment, immunotolerance induced by MUC1 vaccination was reversed by anti-PD-L1 antibodies, a process involving an increased CD80/PD-L1 ratio, promotion of dendritic cell maturation, activation of Th1 and Tc1 cells, and inhibition of Treg cells (115). Similarly, Zhang et al. demonstrated that the co-administration of a MUC1-MBP vaccine with αPD1 antibodies in the B16-MUC1 melanoma model markedly improved anti-tumor efficacy compared to the vaccine alone, mainly by elevating CD8+T cell, Th1, and Tc1 activities, and diminishing the proportion of MDSCs in the tumor microenvironment (116). In research on MUC16, Wang et al. found that individuals with MUC16/CA125 mutations exhibited higher TMB and were associated with an immune-activated microenvironment, elevated interferon gamma (IFNγ) and T cell inflammatory signatures, and enhanced cytotoxic activity. Notably, in male patients, this mutation correlated with better prognosis and higher immune therapy response rates (117). Further genomic data analysis indicated that MUC16 mutations are prevalent in melanoma patients, with such variations closely linked to increased TMB and improved prognosis, potentially by activating immune pathways and enhancing T cell memory functions (118). Additional studies confirmed that mutations in MUC16 in melanoma are closely associated with higher TMB, increased overall survival, and positive responses to anti-CTLA-4 and anti-PD-1 therapies (119).

In studies of urological cancers, MUC1 and MUC16 have been recognized for their significant roles in modulating the immune microenvironment, influencing chemotherapy resistance, and promoting immunosuppression. In clear cell renal carcinoma research, abnormally high expression of MUC1 activates the complement system, colocalizes with the immune marker PTX3, and leads to an increase in M2-type macrophages and a decrease in CD8+ T cells, thus fostering an immunosuppressive microenvironment (120). In castration-resistant prostate cancer (CRPC), MUC1-C protein activation promotes an immunosuppressive environment through the Type II IFN-γ pathway and affects chromatin remodeling, with MUC1-C regulating IDO1, WARS, and PTGES expression, thereby metabolically suppressing the tumor microenvironment and aiding tumor survival and progression, as observed by Hagiwara et al. (121). Yamashita et al. indicated that elevated CA125 expression in bladder cancer is linked to a gemcitabine/cisplatin-resistant tumor microenvironment and decreased survival, associated with regulatory T cells and M2-type macrophages’ infiltration, suggesting a pivotal role in chemotherapy resistance (122). In oral squamous cell carcinoma, Lan et al. found that high expression of MUC1 promotes immune escape, while Porphyromonas gingivalis, by reducing MUC1 and the immunosuppressive factor CXCL17 expression, improves the tumor microenvironment, enhancing the immune system’s ability to clear tumors (123).

**Figure 2:**
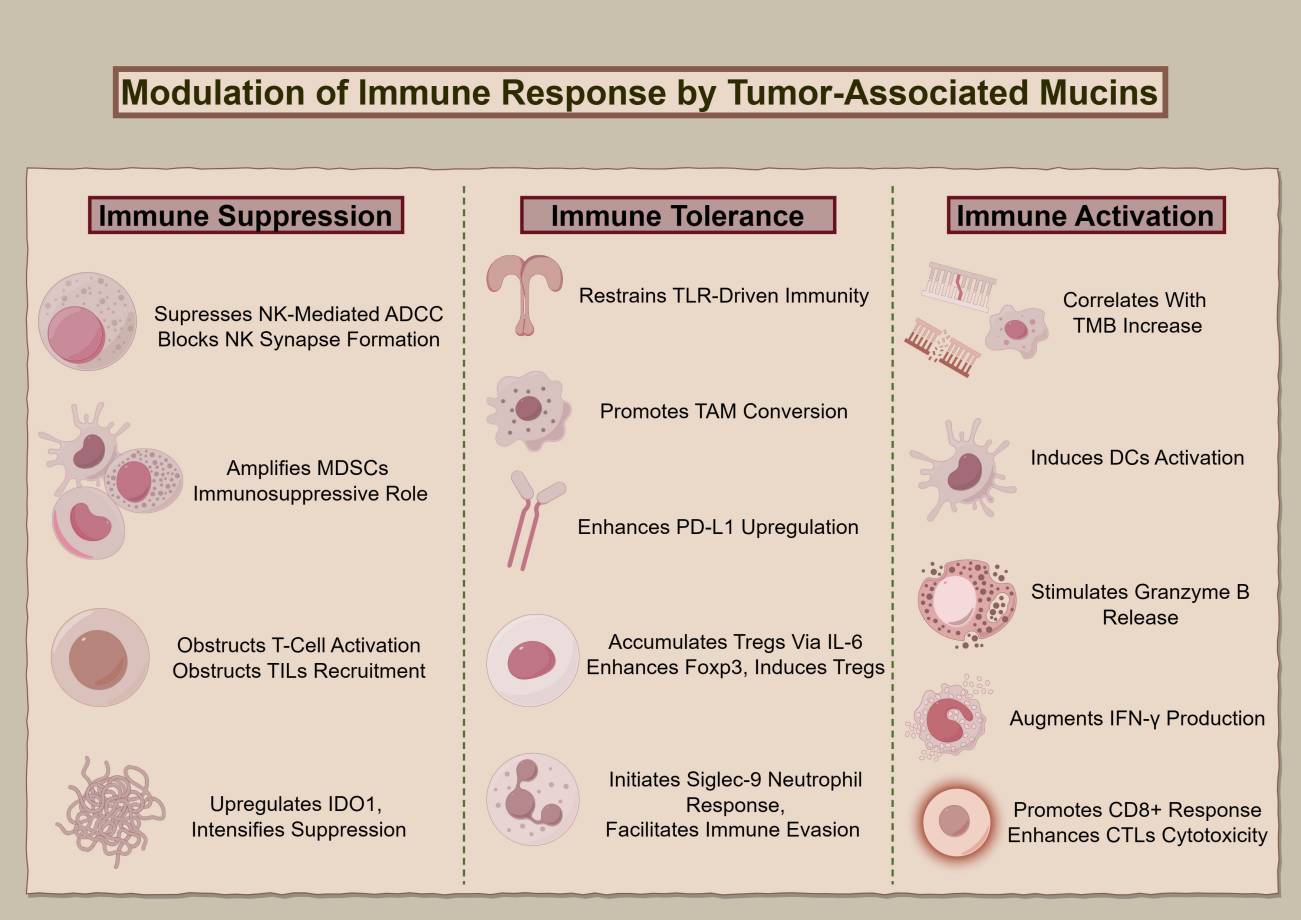
The immunological impact of MUC1 and MUC16. The diagram illustrates the multidimensional functions of MUC1 and MUC16 in immunoregulation, extending from suppression to enhancement of immune functions, highlighting these mucins’ significance as biomarkers for therapeutic interventions.

**Figure 2** delineates MUC1 and MUC16’s regulatory roles regarding immune interactions in oncogenesis, elucidating their contributions to modulating immune responses, from suppression and tolerance to activation, and highlighting their potential as targets for therapeutic intervention.

## Clinical progress of immunotherapies targeting MUC1 and MUC16

The roles and potential of MUC1 and MUC16 in tumor immunotherapy research have garnered widespread attention, especially their application as biomarkers in various cancer treatment strategies. This highlights their potential impact on tumor immune escape mechanisms and therapeutic value.

### Cell-based therapies

#### Preclinical studies

In Posey et al.’s study, CAR T cells targeting the MUC1 Tn glycoform demonstrated significant efficacy against adenocarcinoma, effectively controlling tumor growth and specifically targeting abnormal MUC1 glycosylation in multiple cancer types (124). Additionally, Heukamp et al. demonstrated that clonal CTLs targeting specific epitopes of MUC1 can effectively target tumor cells overexpressing MUC1 without harming normal tissues, highlighting their potential application in cancer immunotherapy (125). Moreover, the integration of anti-tMUC1-CAR T cells with inhibitors targeting IDO1, COX1/2, and Gal-9 has provided a significant strategy to enhance the cytotoxicity of CAR T cell therapy against PDAC cells, addressing the challenge of immunological resistance in pancreatic cancer treatment (126). Hong et al. showed that altering MUC1 epitope structures significantly increases the immunogenicity of pancreatic cancer, stimulating more cytotoxic T cells (CTLs), and indicating potential in enhancing pancreatic cancer immunotherapy (127). Li et al. further demonstrated that shRNA@Fe3O4 magnetic nanoparticles targeting the MUC1-C gene effectively inhibit TNBC progression (128). Parallel to MUC1, the modulation of MUC16 expression has emerged as a key factor in augmenting the response of cancer cells to therapeutic interventions. Morgado et al. found that reducing MUC16 expression through high-dose PPARγ agonists can increase the sensitivity of breast and ovarian cancer tumors to chemotherapy and immune responses (129). Wang et al. developed a dual-specificity T cell engager-modified oncolytic adenovirus (OAd-MUC16-BiTE) targeting MUC16, activating cytotoxic T cells to effectively overcome the immunosuppressive environment in ovarian cancer and enhance therapeutic efficacy (130). In another significant advancement, Mun et al. developed MUC16-specific CAR T cells (4H11) capable of dual attacks against WT1-expressing tumor cells, particularly showing enhanced therapeutic effects in tumors with lower MUC16 expression (131). Chekmasova’s team developed a range of CARs aimed at the extracellular domain of MUC16 (MUC-CD), resulting in T cells equipped with these CARs showing marked cytotoxicity against MUC-CD, thus effectively managing ovarian cancer (132). Further contributing to ovarian cancer therapy, Yue et al. found that an oncolytic adenovirus driven by the MUC16 promoter specifically targets CA-125 positive ovarian cancer cells, offering a new strategy for ovarian cancer treatment (133). Feely et al. developed immunomagnetic nanoparticles targeting MUC16, which efficiently extract circulating tumor cells (CTCs) in liquid biopsies, leveraging the widespread expression of MUC16 in high-grade serous ovarian cancer to provide an effective targeting technique for ovarian cancer detection (134).

#### Clinical trials

Kondo et al.’s clinical study demonstrated that treatment of unresectable or recurrent pancreatic cancer with MUC1 peptide-pulsed dendritic cells (MUC1-DC) and activated cytotoxic T lymphocytes (MUC1-CTL) induced immune responses, achieving complete remission in one patient and stability in five, with an extended average survival time to 9.8 months, without observing severe toxic reactions. This underscores the efficacy and potential of MUC1-based immunotherapeutic strategies in pancreatic cancer treatment (135). Subsequently, Gonzalez et al. noted that peripheral blood mononuclear cells (PBMCs) in ovarian cancer patients exhibit significantly higher CA125 binding compared to healthy donors, providing key insights for the development of new diagnostic markers for ovarian cancer (136). In a Phase I clinical trial, Koneru et al. investigated the safety and initial effectiveness of IL-12-secreting CAR T cells that target MUC-16(ecto+) in patients with recurrent ovarian cancer. The results indicated a reduction in tumor size and symptom relief among patients, alongside enhanced cellular activity in the tumor microenvironment (32).

### Antibodies

#### Preclinical studies

Fang et al. developed a dual-specific antibody (BsAb) targeting MUC1 and CD3, effectively enhancing T cell activation, cytokine release, and cytotoxicity, significantly inhibiting the growth of MUC1-positive tumors in xenograft mouse models (137). Subsequently, Panchamoorthy et al. developed the monoclonal antibody 3D1 targeting the MUC1-C subunit, which exhibits high affinity and specificity for MUC1-C expressing cancer cells, and its monomethyl auristatin E (MMAE) conjugate demonstrated antitumor activity in diverse human cancer models, supporting its clinical potential for treating tumors with MUC1-C overexpression (138). Additionally, Gong et al. developed a new anti-MUC1 antibody with desialylation, enhancing NK cell-mediated ADCC, thus exhibiting increased anti-tumor activity against tumors expressing MUC1-Tn/STn antigens (139). Heuser et al. showed that the C595scFv-Fc-IL2 fusion protein, by binding to MUC1, activates NK cells and enhances their ability to kill MUC1-positive tumor cells, indicating positive implications for clinical treatment of MUC1-positive tumors (140). Concurrently, regarding MUC16, Nicolaides et al. focused on the role of MUC16 (CA125) ADCs, discovering that a specific anti-MUC16 ADC (NAV-001) is significantly effective in eliminating tumor cells, highlighting MUC16’s potential as a therapeutic target (141). Moreover, Garg et al. showed that Meso-TR3, by specifically binding to MUC16-positive tumor cells, significantly promotes tumor cell death, underscoring its therapeutic potential (142).

MUC1 and MUC16, as key biomarkers in gynecological tumors, particularly ovarian and breast cancers, have attracted significant attention for their roles and potential in tumor immunotherapy. In this context, Mony et al. showed that ovarian cancer models expressing MUC1, after anti-PD-L1 antibody treatment, exhibited increased T cell infiltration in tumors and extended survival, indicating MUC1’s potential in ovarian cancer immunotherapy (143). In breast cancer treatment research, MUC1 has emerged as a significant therapeutic target. Kelly et al. discovered that humanized TAB004 (hTAB004), targeting transformed MUC1 (tMUC1), shows notable efficacy in treating TNBC by binding and internalizing tMUC1, effectively inhibiting tumor volume growth and improving patient survival (144). Similarly, Kim et al. isolated specific antibodies against MUC1-C using phage display technology, exhibiting significant inhibition of TNBC cells (145). Isla Larrain et al. indicated that the IgG response to MUC1 in breast cancer patients might contribute to improved prognosis, suggesting that enhancing this natural immune response could be a viable therapeutic strategy (146). Concerning MUC16’s impact, Yeku et al. confirmed the significant cytotoxicity of MUC16 ectodomain-specific bispecific single-chain variable fragments (BiTEDs) against ovarian cancer cells *in vitro*, effectively delaying tumor progression *in vivo*, especially when combined with anti-angiogenic treatment, showing potential for extending survival (147). Additionally, Babeker’s team’s investigation into the fully human monoclonal antibody M16Ab in ovarian cancer immune PET imaging revealed that the 89Zr-DFO-M16Ab conjugate specifically targets MUC16-positive tumor cells, underscoring the potential of fully human antibodies in diagnostic imaging and radiopharmaceutical development (148). Rao et al. identified the oncogenic potential of N-glycosylation sites on the extracellular C-terminal part of MUC16 in ovarian cancer, showing that monoclonal antibodies targeting these sites significantly inhibit ovarian cancer cell invasion and growth of xenograft tumors with MUC16, all driven by an MGAT5-dependent mechanism (149). Furthermore, Marcos-Silva et al. developed the monoclonal antibody 5E11 against a specific peptide segment FNTTER of ovarian cancer MUC16, which specifically recognizes MUC16, leading to the design of a MUC16-based vaccine to stimulate an immune response (150). Stasenko et al. discovered that the high-affinity antibody 14D11 targeting Gal3 inhibits tumor growth in high-grade serous ovarian cancers expressing MUC16, offering a new therapeutic strategy for MUC16-positive cancers (151).

Studies in pancreatic cancer have revealed that MUC1 in both cancer cells and exosomes presents specific dynamic epitopes identifiable by the anti-MUC1 antibody SN-131, which binds distinctively to core 1 type O-glycans on MUC1, as opposed to core 2 types in normal cells, demonstrating the therapeutic potential of targeting these unique O-glycosylation areas on MUC1 in pancreatic cancer cells (152). Schettini et al. demonstrated that coupling anti-MUC1 monoclonal antibodies with CpG ODN significantly enhances the ADCC effect of NK cells, augmenting the immune system’s ability to clear tumor cells, and reducing tumor burden in a mouse model of pancreatic cancer, underscoring the significant role of MUC1 in regulating NK cell functions (153). Additionally, in pancreatic cancer studies on MUC16, Thomas et al. showed that the monoclonal antibody AR9.6 significantly inhibits the AKT and GSK3β pathways activated by MUC16, effectively reducing tumor growth and metastasis and highlighting AR9.6’s potential as a novel immunotherapeutic approach for MUC16-mediated therapy in pancreatic cancer (65). Concurrently, Shah et al. demonstrated that the ch5E6 chimeric antibody against MUC16-Cter displayed anti-tumor effects in pancreatic cancer and non-small cell lung cancer by disrupting tumor growth and signaling, inhibiting tumor cell proliferation, and its interaction with MUC16 and N-cadherin underscored its potential in targeting tumor invasiveness (154).

#### Clinical trials

Silk et al. discovered that patients with colorectal adenomas and cancers had higher anti-MUC1 antibody levels in their blood compared to healthy controls, suggesting that anti-MUC1 immune responses may contribute to tumor development inhibition (155). Advancing the insights into MUC16’s impact on cancer, Akita et al. found that differential expression of the sialyl-Tn antigen on MUC16 can distinguish between ovarian cancer and endometriosis, emphasizing MUC16’s potential importance in ovarian cancer diagnosis and treatment (156). Additionally, Liu et al.’s phase I study assessed the safety and pharmacokinetics of the antibody-drug conjugate DMUC5754A targeting MUC16 in patients with platinum-resistant ovarian cancer (OC) and unresectable pancreatic cancer (PC), observing significant tumor growth inhibition responses in ovarian cancer patients with high MUC16 expression (33).

Given the significance of MUC16 in ovarian cancer treatment, the study of immunotherapies and clinical trials demands special attention. In the 1990s, Wagner et al.’s clinical research already demonstrated the effectiveness of the ACA125 vaccine in inducing immune responses against CA125 in patients with advanced ovarian cancer. In this study, nine patients developed specific immune responses to ACA125, resulting in improved progression-free survival (157). In the MIMOSA trial, led by Battaglia et al., assessed ovarian cancer patients’ response to abagovomab, a MUC16-mimetic antibody. Results showed that patients with stronger immune functions, indicated by the elevated levels and counts of IFN-γ-producing CD8+ T cells, had better responses and improved relapse-free survival (RFS), highlighting the importance of robust immune system functionality in responding to MUC16-targeted treatment (158). Liu et al. conducted a Phase I trial on DMUC4064A, targeting high MUC16 expression in platinum-resistant ovarian cancer. The study demonstrated the drug’s tolerability and initial efficacy, with a notable proportion of patients showing partial response or disease stabilization, suggesting its potential in modulating tumor immunity (159). Similarly, Wang et al.’s Phase II clinical trial showed that in patients with advanced axillary melanoma, apatinib combined with carrelizumab presented positive anti-tumor effects, and mutations in the MUC16 gene were associated with better survival outcomes in patients (160). In a Phase II clinical trial by Nicolaides et al., the effect of the tumor marker CA125 on the immune efficacy of amatuximab (a monoclonal antibody against mesothelin) was explored. Results indicated that patients with baseline CA125 levels below 57 U/mL showed better treatment responses, suggesting that therapeutic strategies should consider patients’ baseline CA125 levels (161).

### Vaccines

#### Preclinical studies

Advancements in vaccine development for MUC1 and MUC16 demonstrate promising outcomes in recent studies. Zhou et al.’s construction of a MUC1 vaccine, combined with aluminum adjuvant and TLR7 agonist, showed significant efficacy in enhancing antibody production and CD8+ T cell immune response against MUC1 (162). Additionally, Panasiuk et al. demonstrated that recombinant chimeric norovirus-like particles (VLPs) combined with MF59 adjuvant effectively induced high-titer IgG antibodies targeting MUC1 in mice, which specifically recognize and target MUC1 on tumor cell surfaces, thereby enhancing immune recognition and clearance of tumors (163). Moreover, Fang et al. revealed that combining MUC1-MBP with BCG stimulates a dual immune response, enhancing Th1-type immunity and the cytotoxic function of MUC1-specific CTLs, while also boosting NK cell activity, effectively inhibiting the growth of MUC1-positive tumor cells (164). Mehrab Mohseni et al. demonstrated that MUC1, a tumor-associated antigen in breast cancer, triggers a robust immune response, marked by the production of anti-glycoprotein serum IgG, IgA, and IFNɣ, effectively mobilizing the immune system against breast cancer proliferation and invasion (165). In Liu et al.’s study, a MUC1 vaccine combined with TLR7 agonist demonstrated significant immune activation and anti-tumor effects in a mouse model of breast cancer, enhancing immune response against MUC1-expressing tumor cells (166). Complementing this, Zhang et al. developed a vaccine containing MUC1 antigen and TLR7 agonist, significantly enhancing the immune response against MUC1-expressing tumors, increasing antibody titers and T cell activity, indicating its potential in breast cancer treatment (167). In immunotherapy research for digestive tract tumors, MUC1’s role is similarly pivotal. Yu et al. demonstrated that modified MUC1 peptides, by enhancing HLA-A0201 mediated T-cell activation, improved the recognition and attack of wild-type MUC1 gastric cancer cells (168). Equally, Guo et al. highlighted the central role of MUC1 in colorectal cancer stem cell vaccine immunotherapy, impacting tumor growth and metastasis, particularly notable in enhancing NK cell toxicity and promoting anti-MUC1 antibody generation (169). Furthermore, in urological tumors, Vang et al. demonstrated, using a MUC1 transgenic mouse model, the potential of specific immune responses to MUC1 for bladder cancer treatment; they found that vaccination with MUC1-specific peptides stimulated a Th1-type cytokine environment and elicited specific T-cell responses to MUC1 (170). Concerning MUC16’s impact on ovarian cancer, Lu et al. have developed an mRNA vaccine targeting neoantigens in MUC16 in breast and ovarian cancers, combined with CD40L and MHC-I targeting domains to enhance dendritic cell antigen presentation efficacy, with computational models predicting that this vaccine could activate IFN-γ and CD8+ T cells, playing a key role in tumor immunotherapy (171). Reinartz et al. demonstrated that fusing IL-6 to an anti-CA125 antibody targeting MUC16 significantly enhanced specific humoral immune responses, effectively inducing the immune system’s response to the CA125 marker (172).

#### Clinical trials

Recent clinical trials have highlighted MUC1 and MUC16’s significant potential as immunotherapy targets in advanced cancer treatment, particularly in various solid tumors. As evidenced by the study by Scheid et al., Tn-MUC1 was established as an efficacious immunotherapeutic target in non-metastatic castration-resistant prostate cancer patients, with the Tn-MUC1 DC vaccine inducing T-cell responses in Phase I/II clinical trials and significantly extending PSADT, indicative of decelerated cancer progression and highlighting the therapeutic promise of Tn-MUC1 in immunotherapy (173). Complementing these findings, innovative vaccine strategies have shown initial success in eliciting specific T-cell responses, contributing to the inhibition of cancer progression and improvement in survival rates. A specific clinical example, such as Tan et al.’s Phase I clinical trial, utilized an adenoviral vector vaccine Ad-sig-hMUC1/ecdCD40L targeting the tumor-associated antigen MUC1 for treating advanced adenocarcinoma. Post-vaccination, patients exhibited improved immune network connectivity, particularly those with stable disease, characterized by a notable increase in CD8 T cells and B cells, highlighting the vaccine’s efficacy in activating multiple branches of the immune system and its significant impact on cancer treatment (174). Similarly, in Gatti-Mays et al.’s Phase I clinical trial, adenovirus 5 vector vaccines ETBX-011, ETBX-051, and ETBX-061, targeting CEA, MUC1, and brachyury, were administered safely to advanced cancer patients, eliciting CD4+ or CD8+ T cell responses to at least one vaccine-encoded antigen in all patients, with 83% demonstrating significant specificity to MUC1 (175). Further, in the same team’s Phase I dose-escalation trial, the BN-CV301 vaccine, incorporating MUC1 and CEA, was shown to be safe and to elicit specific T-cell responses against these tumor antigens in most patients, with significant disease stabilization observed particularly in patients with KRAS-mutated gastrointestinal tumors undergoing anti-PD-L1 therapy, underscoring its potential in immunotherapy (176). Concurrently, Musselli et al. conducted a study on breast cancer patients vaccinated with MUC1 peptide segments conjugated to KLH, revealing that while the immune response to KLH was enhanced, the MUC1-specific T-cell response showed limitations in consistency and intensity (177). In contrast, the phase II ABCSG 34 clinical trial with the tecemotide vaccine targeting MUC-1 failed to demonstrate increased efficacy in the neoadjuvant treatment of breast cancer (178). Meanwhile, Schoen et al. revealed that MUC1 peptide vaccines elicited significant immune responses in a subset of adenoma patients, with 25% showing at least a twofold increase in MUC1 IgG levels after 12 weeks, correlating with reduced adenoma recurrence rates and suggesting MUC1’s role in immune surveillance for intestinal adenoma prevention (179). In advanced NSCLC, the TG4010 vaccine, targeting MUC1 and interleukin-2, indicated enhanced progression-free survival (PFS) in the phase 2b/3 TIME trial, especially when used alongside first-line chemotherapy (180).

**Figure 3:**
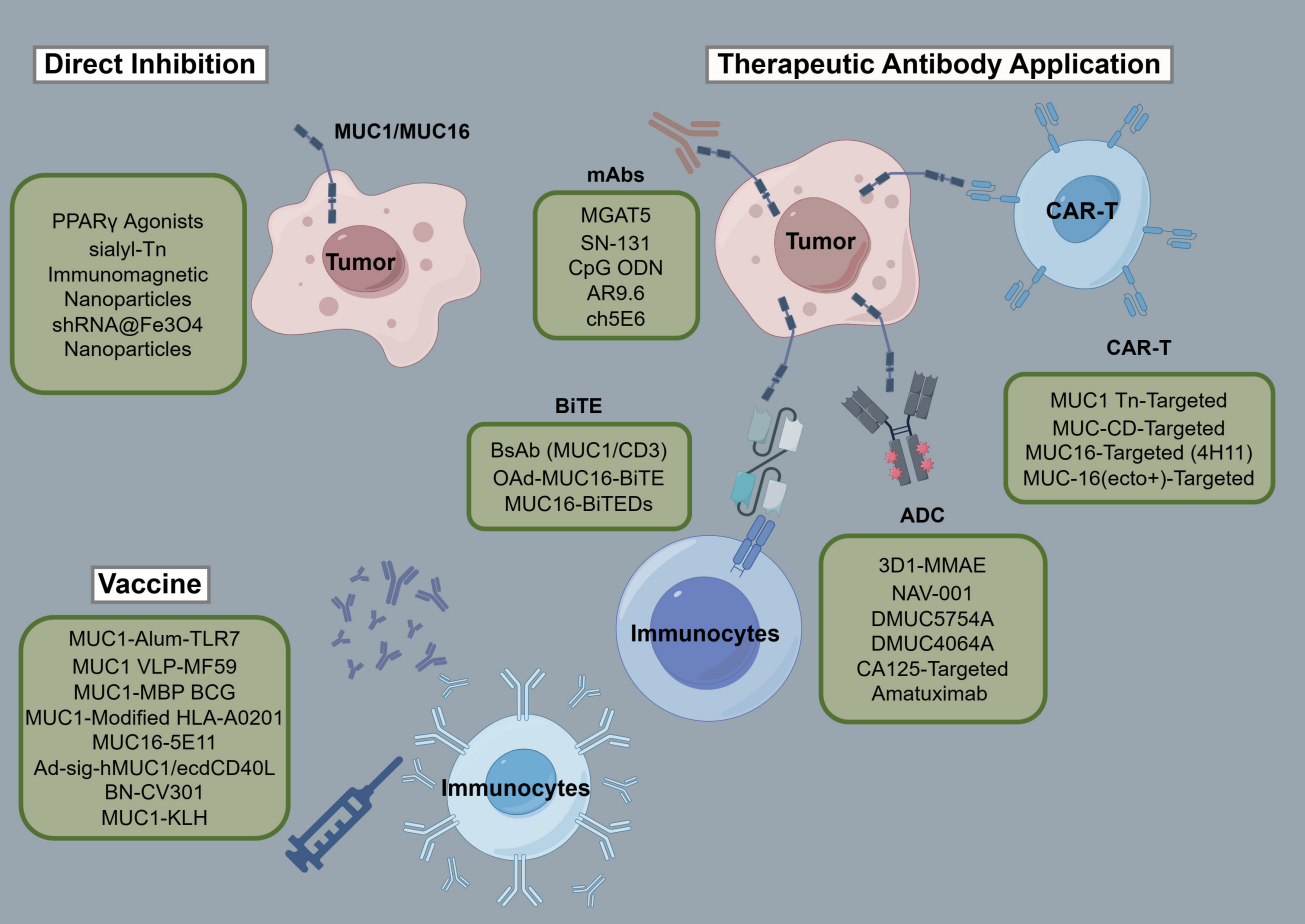
Schematic of immunotherapies targeting MUC1/MUC16. The figure provides a systematic overview of treatments engaging MUC1 and MUC16, including inhibitors, monoclonal antibodies, vaccines, and adoptive therapies, detailing their role in cancer immunomodulation.

**Tables 3** and **4** aggregate the contributions of MUC1 and MUC16 to immunological processes in oncogenesis, alongside their genetic and therapeutic implications. **Figure 3**, corresponding with **Tables 3** and **4**, systematically outlines a variety of immunotherapy methods targeting MUC1 and MUC16, including direct inhibitors, monoclonal antibodies, vaccination strategies, and cellular treatments, thus clarifying their application in cancer therapy. Clinical trials pertinent to these antigens, encompassing patient cohorts and therapeutic outcomes, are detailed in **Table 5**, offering a comprehensive view of current advancements in immunotherapy research.

**Table 3:** MUC1’s immune regulatory roles in carcinogenesis.

Cancer Classification	Immune System Interaction and Immune Cells Responses Impact	Genetic and Molecular Interactions	Clinical Implications and Therapeutic Targets	References
Various Carcinomas	Binds Siglec-9 on Myeloid Cells, Affecting Tumor Environment and Progression	Aberrant Glycosylation (MUC1-ST) Leads to MEK-ERK Activation	Potential Target for Modulating Tumor Microenvironment and Immune Response	(25)
Various Carcinomas	MUC1/CD3 Bispecific Antibody Redirects T Cells Against MUC1-Expressing Tumors	Targets MUC1 for T Cell Activation, Enhancing Cytokine Release and Cytotoxicity	MUC1/CD3 Bispecific Antibody as a Promising Therapeutic Candidate for MUC1-Positive Cancers	(137)
Various Carcinomas	Enhanced NK Cell-Mediated Cytotoxicity via ADCC	Humanized Anti-MUC1 Antibodies Target Under-Glycosylated MUC1 Epitopes (MUC1-Tn/STn) on Malignant Cells; Defucosylation Improves Efficacy	Novel Humanized Anti-MUC1 Antibodies as Promising Candidates for Immunotherapy; Defucosylation Increases Therapeutic Potential	(139)
Advanced Adenocarcinomas (Breast, Lung, Ovary)	Adenoviral-Vector Vaccine (Ad-sig-hMUC1/ecdCD40L) Targets MUC1 and Stimulates Immune Response	Fusion of MUC1 with CD40 Ligand to Enhance Immunogenicity	Phase I Study Shows Safety and Potential Immunotherapeutic Impact of MUC1-Based Vaccine	(174)
Pancreatic and Breast Cancer	MUC1 Repression Enhances MDSC Proliferation and Suppressiveness	Involvement in MDSC Regulation via iNOS, ARG1, TGF-β, c-Myc, and pSTAT3 Pathways	Targeting MUC1 May Reduce MDSC-Mediated Immunosuppression in Tumor Microenvironment	(76)
Triple-Negative Breast Cancer	MUC1-C Upregulates PD-L1, Contributing to Immune Evasion	MUC1-C Elevates PD-L1 Transcription via MYC and NF-κB p65	Targeting MUC1-C as a Potential Immunotherapeutic Strategy	(92)
Triple-Negative Breast Cancer	MUC1-C Activates Immunosuppressive IFN-γ Pathway, Depleting CD8+ T Cells	Regulates TNBC Transcriptome via STAT1, IRF1; Induces IDO1 and COX2/PTGS2	Potential Target for TNBC Treatment, Enhancing Responsiveness to ICIs	(95)
Breast Cancer	MUC1-ST Induces Monocytes to Differentiate into Pathogenic TAMs	Engages Siglec-9, Influencing TAM Phenotype and Function	Targeting MUC1-ST May Counteract TAM-Mediated Immunosuppression and Tumor Progression	(93)
Ovarian Cancer	Anti-PD-L1 Enhances T Cell Response in MUC1-Expressing Tumors	MUC1 Expression Linked to Reduced T Cell Infiltration; Reversed by PD-L1 Blockade	PD-L1 Inhibition as a Strategy for Enhancing Immune Response in MUC1-Positive Ovarian Cancer	(143)
Non-Small Cell Lung Cancer	MUC1-C Upregulates PD-L1, Suppresses CD8+ T-Cell Activation	Influences PD-L1 and IFN-γ Expression; Affects CD8+ TILs Function	MUC1-C as a Target for Modulating Immune Microenvironment	(102)
Non-Small Cell Lung Cancer	Targeted by Tandem CAR-T Cells; Enhanced Efficacy with Anti-PD-1 Antibody	Overexpressed; Co-targeted with PSCA in CAR-T Cell Therapy	Potential for Combined CAR-T Targeting MUC1 and PSCA with Anti-PD-1 Therapy	(104)
Non-Small Cell Lung Cancer	MUC1-C Downregulation Modulates CD8+ T Cell Function; Decreases PD-L1 Expression, Influencing T Cell Apoptosis	Evodiamine Reduces MUC1-C mRNA and Protein; Affects IFN-γ-Induced PD-L1 Expression	MUC1-C as Target for Evodiamine; MUC1-C Overexpressed in Advanced Adenocarcinoma, Notably in Female Non-Smokers	(105)
Colon Cancer	MUC1 Induces Immunosuppression, Increases Treg, MDSCs, and TAMs; PD1/PDL1 Pathway Upregulated	Involves PD1-PDL1 Signaling in Immune Escape	Targeting PD1-PDL1 Pathway as a Therapeutic Strategy for MUC1-Positive Colon Cancer	(108)
Colitis-Associated Colorectal Cancer	MUC1 Promotes Immunomodulation, Increases Pro-tumoral Macrophages, Inhibits CD8+ T Cells	Activates IL-6/STAT3 Axis, Influences Immune Cell Function and Tumor Microenvironment	Inhibiting MUC1 Signaling Could Reduce Inflammation and Tumor Progression	(109)
Colorectal Cancer	MUC1-Expressing CCSC Vaccine Enhances Immune Response; Increases NK Cell Cytotoxicity and Production of IFN-γ, Perforin, Granzyme B	MUC1 Involved in CCSC Maintenance, Tumorigenicity, and Metastasis; Knockdown Impairs Anti-Tumor Efficacy of CCSC Vaccine	MUC1 as a Key Target in CRC Immunotherapy; Potential for Safe and Effective CRC Treatment	(169)
Pancreatic Cancer	Enhanced NK Cell Anti-Tumor Activity via ADCC with CpG-Conjugated Anti-MUC1 Antibody	MUC1 Targeted by HMFG-2 Antibody; Conjugation with CpG ODN Amplifies NK Cell Response	Intratumoral Delivery of CpG-Conjugated Anti-MUC1 Antibody as a Promising Therapeutic Approach	(153)
Cholangiocarcinoma	MUC1 Facilitates Foxp3+ Treg Cell Accumulation in Tumor Microenvironment	Interacts with EGFR; Activates EGFR/PI3K/Akt Signaling Pathway	Targeting MUC1-EGFR Interaction Could Inhibit Tumor Growth and Metastasis	(113)
Castrate-Resistant Prostate Cancer	MUC1-C Induces Immunosuppression via Type II IFN-γ Pathway and Metabolic Suppression	Activates IFN-γ Pathway and Chromatin Remodeling; Regulates IFNGR1, STAT1, IRF1	Inhibiting MUC1-C Could Enhance Immune Response Against CRPC	(121)
Clear Cell Renal Cell Carcinoma	Modulates Immunoflogosis via Complement Activation; Alters Immune Cell Infiltrate	Associated with PTX3 Expression, C1q, CD59, C3aR, C5aR Upregulation; Impacts Mast Cells, M2-Macrophages, IDO1+ Cells, CD8+ T Cells	Potential Therapeutic Target for Immune Modulation	(120)
Melanoma	MUC1-MBP Vaccine Increases Treg Levels, Induces Tolerance; Anti-PD-L1 Antibody Enhances DC Maturation, Decreases Treg Activity	Influences CD80/PD-L1 Ratio on Dendritic Cells; Alters Th1 and Tc1 Cell Activity	Combining MUC1-MBP Vaccine with Anti-PD-L1 Antibody as a Strategy to Overcome Immune Tolerance	(115)
Melanoma	Combination of MUC1-MBP Vaccine and Anti-PD1 Antibody Increases Th1, Tc1 Activity, and Reduces MDSCs	Enhances CD8+ T Cell Mediated Immunity; Targets PD1 Expression on T Cells	Enhanced Anti-Tumor Efficacy with Combined MUC1-MBP Vaccine and Anti-PD1 Treatment	(116)

**Table 4:** MUC16’s immune regulatory roles in carcinogenesis.

Cancer Classification	Immune System Interaction and Immune Cells Responses Impact	Genetic and Molecular Interactions	Clinical Implications and Therapeutic Targets	References
Breast and Ovarian Cancer	MUC16 Overexpression Protects Tumor Cells from Chemotherapy and Immune System	Modulated by PPARγ in Response to Cytokines; Inversely Related to NFκB/p65 Expression	Targeting PPARγ to Reduce MUC16 Expression, Enhancing Tumor Sensitivity to Chemotherapy and Immune Response	(129)
Epithelial Ovarian Cancer	IL-12 Secreting CAR T Cells Target MUC16(ecto), Enhancing Cytotoxicity and Modulating Tumor Environment	MUC16(ecto) Targeted by Genetically Modified T Cells Expressing Chimeric Antigen Receptors	Phase I Trial for MUC-16(ecto)+ Recurrent Ovarian Cancer; Includes Safety Elimination Gene	(32)
Epithelial Ovarian Cancer	Binds to Siglec-9 on NK Cells, B Cells, and Monocytes, Inhibiting Anti-Tumor Immune Responses	csMUC16/sMUC16 Interacts with Siglec-9, Facilitates Adhesion and Metastasis	Potential Target for Interrupting csMUC16-Siglec-9 Mediated Immune Evasion	(36)
Epithelial Ovarian Cancer	MUC16-CAR T Cells Secrete BiTE Targeting WT1; Enhanced Anticancer Activity, Especially in Low MUC16-Expressing Cells	Dual Targeting: Surface MUC16 and Intracellular WT1; Utilizes ESK1 BiTE from TCR Mimic Antibody	Overcomes Heterogeneous Antigen Expression in CAR T Therapy; Promising Strategy for Ovarian Cancer with Varied MUC16 Expression	(131)
Ovarian Cancer	Inhibits Immune Synapse Formation Between NK Cells and Tumor Cells	Heavily Glycosylated Mucin, Carrier of CA125	Implication in Tumor Metastasis and Immune Evasion, Potential Target for Enhancing NK Cell Recognition	(35)
Ovarian Cancer	MUC16 Interacts with Siglec-9 on Neutrophils; Stimulates Inflammatory and Immunosuppressive Neutrophil Phenotype	Correlation with Increased Neutrophil Count and Inflammatory Factors; Influences TNFA, IL6 Pathways, and Immunosuppression-Related Factors	MUC16 as a Modifier of Tumor Microenvironment; Potential Target for Modulating Immune Response	(79)
Ovarian Cancer	MUC16 Suppresses Human and Murine NK Cell and Macrophage Cytolytic Responses	Inhibits NK Cell Cytolysis and Conjugate Formation with Tumor Cells	Targeting MUC16 May Enhance Innate Immune Response Efficacy	(81)
Ovarian Cancer	REGN4018 Bispecific Antibody Bridges MUC16-Expressing Cells with CD3+ T Cells for Enhanced Immune Response	Targets MUC16 on Cancer Cells; Induces T Cell-Mediated Tumor Cell Killing	REGN4018 as a Promising Treatment Option for MUC16-Expressing Advanced Ovarian Cancer	(88)
Ovarian Cancer	MUC16-BiTE Armed Oncolytic Adenovirus Enhances CTL Activity, Overcoming Tumor Immunosuppression	MUC16-BiTE Targets MUC16+ Tumor Cells, Facilitates CTL Cross-Linking and Activation	OAd-MUC16-BiTE as a Promising Therapy, Enhancing Tumor Cell Killing and Reversing the TME	(130)
Ovarian Cancer	MUC16-targeted CAR T Cells Show Cytolytic Activity Against Ovarian Cancer	CARs Specific to MUC16 Extracellular Domain (MUC-CD); Efficacious Against Ovarian Tumor Lines *In Vitro*	Promising Therapeutic Potential for MUC16(+) Ovarian Cancer	(132)
Endometrial Cancer	Mutations Promote Cytotoxic T Lymphocyte Infiltration and Antitumor Immunity	Mutations Correlate with Enriched Cytotoxic Immune Cell Pathways	MUC16 Mutations as a Prognostic Factor; Potential for Immunotherapy Approaches	(100)
Ovarian and Pancreatic Cancers	ADC DMUC5754A Targets MUC16, Exhibits Anti-Tumor Activity	Anti-MUC16 Monoclonal Antibody Conjugated to MMAE	Phase I Safety and Pharmacokinetics Study; Potential Use in MUC16-High Tumors	(33)
Pancreatic Cancer	Promotes Treg Differentiation via Tumor-Derived IL-6	MUC16c Increases IL-6 Expression, Involves PI3K/AKT Pathway	Potential Target for Therapy, Linked to Prognosis and Tumor Burden	(37)
Mesothelioma	CA125/MUC16 Suppresses Immune-Effector Function; Impairs ADCC via Fc-γ-Receptor Engagement	Interferes with Amatuximab Binding and Efficacy; Elevated CA125/MUC16 Levels Associated with Reduced ADCC	Baseline CA125/MUC16 Levels as Biomarker for Amatuximab Response; Potential Stratification Marker in Antibody-Based Therapies for CA125-Producing Cancers	(161)

**Table 5:** Summary of clinical trials on MUC1 and MUC16.

Target Antigen	Clinical Trial Phase	Number of Patients	Therapy Type	Key Findings	References
MUC1	Phase I	20	Cell-based therapy	One complete response, five stable diseases, mean survival 9.8 months	(135)
MUC1	Phase I	148	Antibodies	Higher median anti-MUC1 IgG in abnormal groups (NAA, AA, CRC) than in normals	(155)
MUC16	Phase I	77	Antibodies	DMUC5754A shows acceptable safety and anti-tumor activity in MUC16-high patients	(33)
MUC16	Phase I	16	Antibodies	ACA125 triggered immune responses against CA125 in 56% of patients, with improved survival in responders	(157)
MUC16	Phase I	111	Antibodies	Abagovomab efficacy linked to higher IFN-γ CD8+ T cells, with no similar trend in placebo	(158)
MUC16	Phase I	65	Antibodies	DMUC4064A was well-tolerated, with 42% clinical benefit rate and median progression-free survival of 3.9 months. Notably, a quarter of patients showed a partial response at higher doses	(159)
MUC1	Phase I/II	17	Vaccines	The vaccine was safe and induced significant T-cell responses. 11 of 16 evaluable patients showed improvement in PSA doubling time, suggesting biological activity	(173)
MUC1	Phase I	21	Vaccines	Vaccine demonstrated good tolerability and induced noticeable immunome changes in patients	(174)
MUC1	Phase I	10	Vaccines	The vaccine was safe, with all patients developing T-cell responses to at least one antigen. 83% developed MUC1-specific T cells	(175)
MUC1	Phase I	12	Vaccines	BN-CV301 showed safety, induced targeted immune responses, and led to positive clinical outcomes, notably in KRAS-mutant gastrointestinal tumors	(176)
MUC1	Phase II	400	Vaccines	Tecemotide was safe but did not notably improve treatment efficacy in early breast cancer	(178)
MUC1	Phase I/II	103	Vaccines	25% of MUC1 vaccine recipients had heightened MUC1 IgG levels, and immune responders showed a reduced rate of adenoma recurrence compared to placebo	(179)
MUC1	Phase IIb/III	222	Vaccines	TG4010 enhanced progression-free survival relative to placebo. TrPAL biomarker showed promise in predicting responses	(180)

## Conclusions and perspectives

In the pursuit of furthering MUC1 and MUC16 research, the current understanding of their role in tumor biology and the tumor microenvironment presents a foundation for future exploration. The refinement of therapies such as vaccines, ADCs, and CAR-T cell treatments, alongside the elucidation of these proteins in therapeutic resistance, constitutes a critical research trajectory. The integration of precision medicine, leveraging genomic and proteomic technologies, is essential for tailoring patient-specific therapies. Furthermore, innovative clinical trial designs are imperative for the swift evaluation and enhancement of emerging treatments. As new strategies are introduced, their long-term safety and efficacy must be rigorously assessed, underscoring the need for a multidisciplinary approach in advancing cancer treatment.

## Author contributions 

XC: Formal analysis, Methodology, Writing – original draft, Writing – review & editing. IS: Data curation, Validation, Writing – review & editing. MY: Investigation, Resources, Writing – review & editing. JT: Investigation, Resources, Supervision, Writing – review & editing. XY: Conceptualization, Funding acquisition, Supervision, Writing – review & editing.
